# Ability of community pharmacists to independently perform CLIA-waived testing – A multistate legal review

**DOI:** 10.1016/j.rcsop.2021.100024

**Published:** 2021-05-08

**Authors:** Joseph Doucette, Joseph Lavino

**Affiliations:** a Massachusetts College of Pharmacy and Health Sciences University, Candidate for Doctor of Pharmacy-Accelerated '21, 1260 Elm Street, Manchester, NH 03101, USA; b Massachusetts College of Pharmacy and Health Sciences University, 179 Longwood Ave, Boston, MA 02115, USA

**Keywords:** Pharmacist, CLIA, CLIA-waived

## Abstract

The scope of practice of community pharmacy has continued in its evolution into a clinically focused profession. A critical component to pharmacist clinical offerings in the United States is the performance of Clinical Laboratory Improvement Amendments (“CLIA”) waived tests. However, state laws and regulations greatly influence a pharmacist's ability to perform a CLIA-waived test. Based on this variability and lack of clarity in state laws and regulations, a legal review was performed, which focused on the following themes: 1) states which currently allow a pharmacist to independently perform CLIA-waived testing; 2) of those states, which states impose restrictions on the scope of CLIA-waived tests that may be independently performed by a pharmacist; and 3) states that impose restrictions on the ability of a pharmacist to independently perform CLIA-waived testing. Federal laws already exist that allow individuals, pursuant to certification, to perform CLIA-waived testing. This allowance presumably includes pharmacists if they follow federal law. However, some states regulate and restrict this area for pharmacists, contrary to these federal laws. States may see an improvement in patient care and further expand the practice of pharmacy by limiting restrictions on pharmacists to perform CLIA-waived tests and following federal allowances.

## 1 Background

### 1.1 The clinical laboratory improvement amendments of 1988 (CLIA)

CLIA refers to the Clinical Laboratory Improvement Amendments of 1988, which specifically exist to address how a laboratory handles any analyte from a human specimen.^1^ Within the law, certain exemptions exist whereby a laboratory would not need to comply with aspects of the law. For instance, these exemptions include laboratories that utilize any human specimen for research, forensics purposes, or those under the jurisdiction of the federal government.^2^ In addition to these exemptions, different types of certification are currently available for any laboratory wishing to perform CLIA testing. These include a certificate of compliance, certificate for provider-performed microscopy (PPM), certificate of accreditation, certificate of registration and certificate of waiver.^1^ Each of these certificates differ by allowing the laboratory to perform a variety of different testing, largely based upon complexity. A community pharmacist could optimize their clinical services through the acquisition of a CLIA certificate of waiver with the goal of achieving improvements in health, experience of care, and costs.^2^^,^^3^^,^^103^

### 1.2 CLIA certificate of waiver

The CLIA certificate of waiver is applied to testing based upon several parameters. Specifically, the test must be cleared by the FDA for home use, apply methods that are so accurate and intuitive that the likelihood of error is negligible and, should the test be performed incorrectly, the chance of patient harm would pose no reasonable risk.^3^ Testing of this variety encompasses a lengthy list, but some examples include dipstick or tablet reagent urinalysis, fecal occult blood tests, ovulation tests, hemoglobin tests and blood glucose screenings.^3^ A study by Klepser et al. found community pharmacies in the United States to be the fourth largest entity to hold certificates of waiver to perform CLIA-waived testing.^4^ Approximately 17.94% of all pharmacies were found to hold certificates of waiver, which indicates a large potential for growth, and it was estimated that community pharmacies could easily become the second largest entity to offer CLIA-waived testing.^4^

Federal statutes set clear expectations and requirements for any individual hoping to obtain a CLIA certificate of waiver and begin testing.1., 2., 3. However, legislation or regulation at the state level often complicates this simple narrative and creates a great deal of confusion and interpretation variability for pharmacists. Many states possess statutes or regulations that either bar a pharmacist from performing CLIA-waived testing outright or the language itself creates a great deal of obscurity as to whether a pharmacist could perform CLIA-waived testing in a compliant manner with state law or regulations.

Imagine a situation in which a patient enters a pharmacy seeking a CLIA-waived test to determine the presence of the influenza virus, a simple flu test. However, the pharmacist on duty happens to be practicing in a state which bars CLIA-waived testing; or alternatively, the state laws or regulations allow for certain types of testing, such as testing pursuant to drug therapy. In either case, the simple flu test would not be provided to the patient by the pharmacist. Given how the very definition of a CLIA-waived test is one that must be so simple as to remove error nearly entirely from the equation, it is questionable as to why state laws and regulations impose any further restrictions, confusion or ambiguity upon pharmacists in this regard.

## 2 Research results and discussion

A survey of laws and regulations was performed to determine the status of CLIA-waived testing within each state. States were looked at through the lens of a pharmacist having the ability to independently perform a CLIA-waived test, assuming all appropriate paperwork and licensure was in place. The study included state and federal pharmacy statues and regulations, state department of health statues and regulations and Covid-19 state allowances via executive orders or Board of Pharmacy guidance. Each jurisdiction was given one of four designations based upon the question of whether a pharmacist may independently perform any CLIA-waived test, which is summarized in Table 1.

**Table 1:** Jurisdictions and Respective Independent CLIA-Waived Testing Status as Performed by a Pharmacist. The question is, may a pharmacist independently perform a CLIA-waived test per state laws, regulations or guidance?

Designation	Jurisdictions
Yes	Alabama,^5^^,^^6^ Alaska,^7^^,^^8^ California,9., 10., 11., 12. Colorado,13., 14., 15. Delaware,^16^ District of Columbia,^17^ Georgia,^18^ Idaho,^19^ Louisiana,^20^ Maryland,^21^ Minnesota,^22^ New Mexico,^23^^,^^24^ North Carolina,^25^ North Dakota,26., 27., 28. Ohio,^29^ Oregon,^30^ Pennsylvania^31. 32. 33^, Rhode Island,^34^^,^^35^ Tennessee,36., 37., 38. Virginia,39., 40., 41. Washington^42,^ 43, Wyoming^44^
No	Arizona,^45^^,^^46^ Connecticut,^47^^,^^48^ Florida,50., 51., 52. Hawaii,^53^^,^^54^ Illinois,55., 56., 57. Iowa,58., 59., 60. Kentucky,61., 62., 63. Massachusetts,^64^^,^^65^ Missouri,^66^^,^^67^ New Jersey,^68^ Utah^69^^,^^70^
Unclear	Indiana,^73^ Maine,^74^^,^^75^ Nevada76., 77., 78.
Silent	Arkansas,^79^^,^^80^ Kansas,^81^^,^^82^ Michigan,^83^ Mississippi,^84^ Montana,^85^^,^^86^ Nebraska,^87^^,^^88^ New Hampshire,^89^^,^^90^ New York,^91^^,^^92^ Oklahoma,^93^ South Carolina,^94^ South Dakota,^95^ Texas,^96^ Vermont,^97^^,^^98^ West Virginia,^99^ Wisconsin^100^

### 2.1 Pharmacists may independently perform CLIA-waived testing

Of the myriad of states that have fallen into the category of ‘yes’ in Table 1, several of them are far more complex than a simple allowance to perform any CLIA-waived test. As can be seen in Table 2, some jurisdictions varied in the methodology they applied to allow pharmacists to perform CLIA-waived testing. If laws, regulations, or Board of Pharmacy guidance are deemed necessary by a state, North Carolina may be looked at as a good example. In that case, the Board of Pharmacy has made it explicitly clear to look to the federal CLIA regulations.^25^ California is a unique jurisdiction, as it allows for a varying degree of allowable testing, depending upon a pharmacist's work setting.9., 10., 11., 12. Pharmacists in a community setting are confined to a mere three tests that may be performed.9., 10., 11., 12. Similarly, Delaware has allowances for blood capillary testing, however other types of CLIA-waived tests are excluded.^16^

**Table 2:** Jurisdictions Which Allow a Pharmacist to Independently Perform CLIA-Waived Testing (Section 2.1).

Jurisdiction	Summary
Alabama	Per Covid-19 testing guidance, pharmacies can perform **any** CLIA waived test with proper CLIA certification. Additionally, the guidance refers to the Alabama Dept. of Public Health CLIA standards.^6^
Alaska	The definition of pharmacy practice includes the provision of those acts or services necessary to provide pharmaceutical care and pharmaceutical care is defined as patient care services intended to achieve outcomes related to the cure or prevention of a disease; this presumably includes CLIA waived tests. Refer to the Alaska Dept. of Public Health CLIA standards.^7^
California	Pharmacists in a community setting may perform CLIA-waived testing, per patient request, concerning blood glucose, HbA1c and/or cholesterol; pharmacists in a healthcare facility setting may perform a larger array of drug therapy-related CLIA-waived tests; certain statutes mandate the administrator of a CLIA-waived test must be a lab director, but other statutes exempt pharmacists from this requirement.9., 10., 11., 12.
Colorado	A pharmacist can perform CLIA-waived tests pursuant to pharmaceutical care. The definition of pharmacy practice includes the provision of those acts or services necessary to provide pharmaceutical care in all areas of patient care and pharmaceutical care is defined as patient care services intended to achieve outcomes related to the cure or prevention of a disease. This presumably includes CLIA waived tests. Previously, pharmacists were required to meet minimum qualifications for drug therapy management under 3 CCR 719–1:6.00.30. However, this has since been repealed.13., 14., 15.
Delaware	The pharmacy practice definition allows a pharmacist to perform and interpret capillary blood tests to screen and monitor disease risk factors or facilitate patient education.^16^
District of Columbia	The pharmacy practice definition allows a pharmacist to conduct health screenings, including obtaining finger-stick blood samples, which is a CLIA waived test.^17^
Georgia	The pharmacy practice definition allows a pharmacist to perform and interpret capillary blood tests to screen and monitor disease risk factors or facilitate patient education.^18^
Idaho	A pharmacist may perform any CLIA-waived test. The pharmacy practice definition allows a pharmacist to perform a test that is used to guide diagnosis or clinical decision-making and are waived under the federal clinical laboratory improvement amendments of 1988.^19^
Louisiana	A pharmacist may perform any CLIA-waived test. Guidance from the board of pharmacy indicates that a pharmacist may perform any laboratory testing bearing a CLIA designation of waived or moderately complex.^20^
Maryland	The MD Dept of Health regulations stipulate that a pharmacist may be a lab director for CLIA waived tests.^21^
Minnesota	A pharmacist may perform CLIA-waived tests Pharmacy practice definition allows for the performance of CLIA-waived test, pursuant to monitoring drug therapy.^22^
New Mexico	It is the Board's long-standing position that pharmacists may administer CLIA waived tests per COVID-19 guidance.^23^^,^^24^
North Carolina	Based upon guidance from the North Carolina Board of Pharmacy, a pharmacist may perform any CLIA-waived test.^25^
North Dakota	A pharmacist may perform CLIA-waived tests based on a listing regulated by the Board of Pharmacy.26., 27., 28.
Ohio	A pharmacist may perform any CLIA-waived testing.^29^
Oregon	A pharmacist may perform any CLIA-waived test.^30^
Pennsylvania	A pharmacist may order and perform laboratory examinations and procedures for covid-19, influenza and streptococcal infections. They may order and perform other diagnostic tests necessary in the management of drug therapy with a Collaborative Practice Agreement.31., 32., 33.
Rhode Island	A pharmacist may perform any CLIA-waived test.^34^^,^^35^
Tennessee	Pharmacists are authorized to conduct and assist patients with tests approved for home use. Pharmacists may order CLIA waived tests pursuant to a Collaborative Practice Agreement.36., 37., 38.
Virginia	The Virginia Board of Pharmacy has a longstanding position that the performance of CLIA waived testing is within the scope of practice of pharmacy. Tests must be administered in accordance with federal CLIA requirements. Of note, the definition of collaborative agreements includes procedures related to drug therapy, including lab tests. However, this context likely refers to initiation, modification, or discontinuation of drug therapy.39., 40., 41.
Washington	Per COVID-19 Frequently Asked Questions, pharmacists can administer tests under their scope of practice, which includes the monitoring of drug therapy and use.^42^^,^^43^
Wyoming	A pharmacist may perform Medication Therapy Management services, without a Collaborative Practice Agreement, which includes the ordering, or performing laboratory assessments, and evaluating the response of the patient to therapy, as it directly relates to Medication Therapy Management.^44^

These differences highlight a glaring issue. What exactly makes one CLIA-waived test allowable, while others must be forbidden or stringently monitored, if the very nature of all CLIA waived tests are approved due to being so simple as to remove error nearly entirely from the equation?

Similarly, there are states that allow for pharmacists to perform certain CLIA-waived tests; however, those tests may only be directly related to drug therapy management. Recalling back to the example of an individual inquiring about testing for the presence of influenza, a pharmacist practicing in a jurisdiction with language alluding to drug therapy management would not be able to help this individual because the intent of the test is to monitor for a condition or disease rather than a drug therapy monitoring parameter.

### 2.2 Pharmacists may not independently perform CLIA-waived testing

There are states that impose laws or regulations that negate a pharmacist's ability to perform CLIA-waived testing because of a requirement to do so through collaboration with a physician. Collaborative practice agreements most often embody a delegation by a physician to a pharmacist whereby the pharmacist may implement, modify, or discontinue drug therapy. The scope and capabilities of collaborative practice agreements are also highly dependent on state specific laws and regulations and each state tends to have different terminology used such as drug therapy management, collaborative drug therapy management or drug therapy protocols. For example, Massachusetts defines collaborative practice to include agreement language that limits the age group of patients, disease states that may be managed and how a pharmacist may implement a patient's current drug regimen.^71^ New Hampshire includes many of the same elements within their definition, but also adds which types of laboratory testing may be ordered by the pharmacist and under what circumstances the pharmacist must contact the physician.^72^

The addition of the collaborative practice agreement language to some of the jurisdictions mentioned in Table 3 appear to be justified from the standpoint of a pharmacist utilizing a CLIA waived test as an adjunct to the initiation, modification or discontinuation of drug therapy incorporated into the agreement, however the generalized language also appears to undermine the pharmacist's ability to independently perform CLIA-waived testing outside the scope of a collaborative practice agreement.

**Table 3:** Jurisdictions Which Do Not Allow a Pharmacist to Independently Perform CLIA-Waived Testing (Section 2.2).

Jurisdiction	Summary
Arizona	The definition of pharmacy practice allows for drug therapy monitoring or management, however drug therapy management, which includes lab tests that may be ordered, requires a protocol with a practitioner. Therefore, there is no explicit independent RPh testing authority.^45^^,^^46^
Connecticut	No specimen shall be accepted for analysis or collected by an owner or an employee of the laboratory except when requested by a licensed physician or other licensed person authorized by law to make diagnoses.^47^^,^^48^
Florida	A pharmacist must enter a written protocol from a licensed physician to perform testing concerning minor, nonchronic health conditions, which includes influenza, streptococcus, lice, skin conditions, such as ringworm and athlete's foot and minor, uncomplicated infections.49., 50., 51., 52.
Hawaii	The pharmacy practice definition allows for drug therapy related lab tests by a pharmacist in collaboration with a physician.^53^^,^^54^
Illinois	The pharmacy practice definition allows for medication therapy management, which involves the review of a patient's lab values, however it does not grant authority for a pharmacist to perform the test and a practitioner agreement is required. Additionally, the department of health authorized private individuals to perform 1) blood total cholesterol testing and 2) blood glucose testing by the finger stick method, pursuant to a practitioner protocol. Allowance for COVID-19 testing.55., 56., 57.
Iowa	Regulations allow for only drug therapy management related lab tests by a pharmacist in collaboration with a physician.58., 59., 60.
Kentucky	While pharmacies are exempt from operating as a laboratory if they hold a valid CLIA certification, pharmacists are limited to assisting a patient with the use of CLIA-waived tests available from the facility's stock or inventory, in connection with testing and treatment of patients covered under collaborative care agreements.61., 62., 63.
Massachusetts	Regulations allow for only drug therapy management related lab tests by a pharmacist in collaboration with a physician.^64^^,^^65^
Missouri	A pharmacist may order OR perform lab testing pursuant to a written medication services protocol from a physician.^66^^,^^67^
New Jersey	A pharmacist may order and perform waived testing only pursuant to a collaborative practice agreement.^68^
Utah	A pharmacist may order, evaluate, and perform waived testing only pursuant to a drug therapy management protocol.^69^^,^^70^

### 2.3 Pharmacists may, or may not, independently perform CLIA-waived testing

There are certain states that were simply ‘unclear’ because these states have provided conflicting information, which are summarized in Table 4. For example, Indiana state law does not address CLIA-waived testing, however the Indiana State Department of Health had released guidelines, which specify the entities that can pursue registration to perform CLIA-waived testing, excluding pharmacies.^73^ Maine presents a conflict between their statute, which states that a pharmacist may only perform CLIA-waived testing pursuant to a collaborative practice agreement and documentation released by the attorney general of the state, which states that a pharmacist may perform any CLIA-waived testing.^74^^,^^75^ Nevada has put three separate statutes and regulations in place, and all seem to directly contradict one another.76., 77., 78. One claims to require a collaborative practice agreement, another infers that any CLIA-waived testing may be performed and the final regulation states that only those tests utilizing either a fingerstick, nasal or oral swab are appropriate.76., 77., 78.

**Table 4:** Jurisdictions Which May, or May Not, Allow a Pharmacist to Independently Perform CLIA-Waived Testing (Section 2.3).

Jurisdiction	Summary
Indiana	No legislation exists which specifically bars, or authorizes pharmacists to perform CLIA-waived testing, but the Indiana State Department of Health has released guidelines for CLIA-waived testing.^73^
Maine	A pharmacist must pursue a collaborative practice agreement to perform CLIA-waived testing, but the attorney general has released documents stating pharmacist may perform any CLIA-waived testing.^74^^,^^75^
Nevada	The practice of pharmacy definition states a pharmacist must pursue a collaborative practice agreement to perform CLIA-waived testing. However, separate language states that a pharmacist may perform any CLIA-waived testing, pursuant to federal standards and licensure. Finally, a third reference states that a pharmacist may only perform CLIA-waived testing categorized by collection method: fingerstick, nasal or oral swab.76., 77., 78.

A pharmacist operating within these jurisdictions may not possess the confidence to know with certainty, based on their interpretation of the applicable laws and regulations, as to whether performing a CLIA-waived test is appropriate, or if it could place them at risk of discipline by their licensing authority.

### 2.4 State laws, regulations and board of pharmacy guidance are silent concerning CLIA-waived testing

Jurisdictions which exist within the ‘silent’ designation simply have no mention of CLIA-waived testing within their pharmacy related laws, regulations, and guidance. “Permissionless innovation” is a concept which means to allow for improvement of current systems through market determination.^101^ The idea is not to have zero regulations, as that would be chaotic and lack accountability.^101^ Instead, it enables providers to work at their optimal level of comfort within their scope of practice, while still complying with minimal regulations that allow providers to practice at the top of their license and education.^101^

The jurisdictions in Table 5 cover the array of exceptions encountered within the ‘silent’ designation. A pharmacist practicing in one of these jurisdictions would be left without knowing how to approach a CLIA-waived test. Given that a multitude of states have adopted ample regulations concerning the matter, and given that the practice of pharmacy as a whole is a heavily regulated profession, some pharmacists may feel it is better to play it safe, and simply not perform any sort of CLIA-waived testing. However, some pharmacists, who may adopt a permissionless innovation stance, may see fit to perform the testing because the laws or regulations do not state they can't perform this testing.^101^ Regardless of the viewpoint one may take, states such as these may ease a pharmacist's concern, over whether they are acting in a compliant manner, by simply providing guidance to the industry that references the federal CLIA regulations.

**Table 5:** Jurisdictions Which are Silent Concerning CLIA-Waived Testing (Section 2.4).

Jurisdiction	Summary
Arkansas	The laws and rules do not address CLIA waived testing. The definition of pharmacy practice does not appear to provide a direct allowance. Lastly, the disease state management regulations do not specifically address lab testing.^79^^,^^80^
Kansas	The laws and rules do not address CLIA waived testing. The definition of pharmacy practice does not appear to provide a direct allowance. Lastly, the collaborative practice statute does not specifically address lab testing.^81^^,^^82^
Michigan	The laws and rules do not address CLIA waived testing. The definition of pharmacy practice does not appear to provide a direct allowance. Lastly, the collaborative practice statute does not specifically address lab testing.^83^
Mississippi	The laws and rules do not address performing CLIA waived testing. The definition of pharmacy practice does allude to ORDERING pursuant to a protocol, however, performing tests are not addressed.^84^
Montana	The laws and rules do not address performing CLIA waived testing. The clinical pharmacist qualifications statute does allude to ORDERING pursuant to a CPA, however, performing tests are not addressed.^85^^,^^86^
Nebraska	The laws and rules do not address CLIA waived testing. The definition of pharmacy practice does not appear to provide a direct allowance. Lastly, the collaborative practice statute does not specifically address lab testing.^87^^,^^88^
New Hampshire	The laws and rules do not address performing CLIA waived testing. The definition of pharmacy practice does allude to ORDERING pursuant to a CPA, however, performing tests are not addressed.^89^^,^^90^
New York	The laws and rules do not address performing CLIA waived testing. The definition of pharmacy practice does allude to ORDERING pursuant to a CPA, however, performing tests are not addressed.^91^^,^^92^
Oklahoma	The laws and rules do not address CLIA waived testing. The definition of pharmacy practice does not appear to provide a direct allowance. Lastly, the collaborative practice statute does not specifically address lab testing.^93^
South Carolina	The laws and rules do not address CLIA waived testing. The definition of pharmacy practice does not appear to provide a direct allowance. Lastly, the collaborative practice statute does not specifically address lab testing.^94^
South Dakota	The laws and rules do not address CLIA waived testing. The definition of pharmacy practice does not appear to provide a direct allowance. Lastly, the collaborative practice statute does not specifically address lab testing.^95^
Texas	The laws and rules do not address CLIA waived testing. The definition of pharmacy practice does not appear to provide a direct allowance. Lastly, the collaborative practice statute does not specifically address lab testing.^96^
Vermont	The laws and rules do not address CLIA waived testing. The definition of pharmacy practice does not appear to provide a direct allowance. Of note, CLIA waived testing, while not directly addressed, appears to fall under the definition of clinical pharmacy practice. However, that does not always require a CPA unless there is initiation, modification, or discontinuation of drug therapy.^97^^,^^98^
West Virginia	The laws and rules do not address performing CLIA waived testing. The definition of pharmacy practice does allude to ORDERING pursuant to a CPA, however, performing tests are not addressed.^99^
Wisconsin	The laws and rules do not address CLIA waived testing. The definition of pharmacy practice does not appear to provide a direct allowance. Lastly, the collaborative practice statute does not specifically address lab testing.^100^

## 3 Recommendation and conclusion

The information gathered through this multistate survey has brought many points to light. For those states that allow pharmacists to perform CLIA-waived tests but restrict the type of tests, it would benefit the patient and profession to defer to the federal CLIA regulations and remove those restrictions. Similarly, for those states that do not allow a pharmacist to perform a CLIA-waived test or restrict those tests to collaborative practice agreements, expansion to meet the federal allowances would be beneficial to the public. The very definition of a CLIA-waived test negates error, and even in cases where an error occurs, the risk to patient harm is negligible.^3^ Most of the scrutiny that any of the jurisdictions have imposed, on top of the already present federal regulations, appears on its face to be unnecessary and confusing. For those states that are unclear or silent on the matter, guidance from the Board of Pharmacy clarifying a pharmacist's ability to perform CLIA-waived testing, in compliance with federal regulations, would be helpful to the industry.

The simplification of this matter seems more prevalent now more than at any other time. The Covid-19 pandemic has shed light on many things, but notably how pivotal the role of the pharmacist has become in the delivery of healthcare due to being one of the most accessible members of the healthcare industry. Covid-19 testing has been broadly allowed and occurred regularly in community pharmacies for a reason; they are accessible, and the staffs are well trained to perform these functions. Utilizing them for one type of testing, such as Covid-19 testing, only demonstrated that pharmacies may be used as centers for all CLIA-waived testing.

The practice of pharmacy and patients alike would greatly benefit by having states give broad allowances to pharmacists to perform any CLIA-waived test, in compliance with federal regulations. States may take different approaches to reach that goal. For example, Idaho has adopted a minimalist regulatory scheme in general, but it works to achieve the goal of allowing a pharmacist to perform any CLIA-waived test.^19^ North Carolina is another example of how to proceed, but in a different way: through Board of Pharmacy guidance.^25^ One of the state policies of the National Alliance of State Pharmacy Associations is to promote pharmacist prescribing based on the results of a rapid diagnostic test for strep or flu across the Unities States.^102^

Thinking back on the individual who walks into a community pharmacy for an influenza test, the result of that interaction will vary across the states and jurisdictions. An unfortunate patient encounter may come from a state that allows CLIA-waived testing, but only pursuant to drug therapy management. In that case, even though the pharmacist can perform CLIA-waived testing, an influenza test lies out of bounds. States with limitations such as these may be isolating entire populations of patients that can be served, and every community pharmacist should be unrestricted to help the greatest number of patients, within their scope of practice.

## Disclaimer

The views expressed in this manuscript are those of the authors alone, and do not necessarily reflect those of their respective employers or universities.

## Declaration of Competing Interest

The authors declare that they have no known competing financial interests or personal relationships that could have appeared to influence the work reported in this paper.
